# Large Vaginal and Bladder Calculi in a Woman With Previous Operation of Bladder Exstrophy: A Case Report

**DOI:** 10.5812/numonthly.13637

**Published:** 2013-12-25

**Authors:** Mahmoud Tavakkoli, Alireza Ghoreifi

**Affiliations:** 1 Department of Urology, Imam Reza Academic Hospital, Mashhad University of Medical Sciences, Mashhad, IR Iran

**Keywords:** Vagina, Calculi, Bladder Exstrophy, Urolithiasis

## Abstract

This is to report the case of a huge vaginal stone, and bladder calculi in a 26-year-old woman with previous operation of bladder exstrophy. It seems that the vaginal stone was secondary to the remaining wire used in her previous reconstructive surgery for pelvic closure 20 years ago and now surgery is performed to remove the vaginal and bladder stones.

## 1. Introduction

Vaginal stones are very rare and often misdiagnosed as bladder calculi on plain radiography (1). Urethrovaginal fistula is the main cause of vaginal calculi and the other etiologies are extremely rare (2). Primary vaginal calculi result from the stasis of urine in the vagina, but secondary vaginal calculi originate from crystallization of urinary components around a foreign body in the vagina (3). Bladder exstrophy is a rare condition occurring one in 25,000-50,000 live births (4). A Medline search revealed only one case of vaginal calculi concurrent with bladder exstrophy (5). Here we presented a case of large vaginal and bladder stones in a 26-year-old woman with previous operation of bladder extrophy.

## 2. Case Report

**Figure 1.**
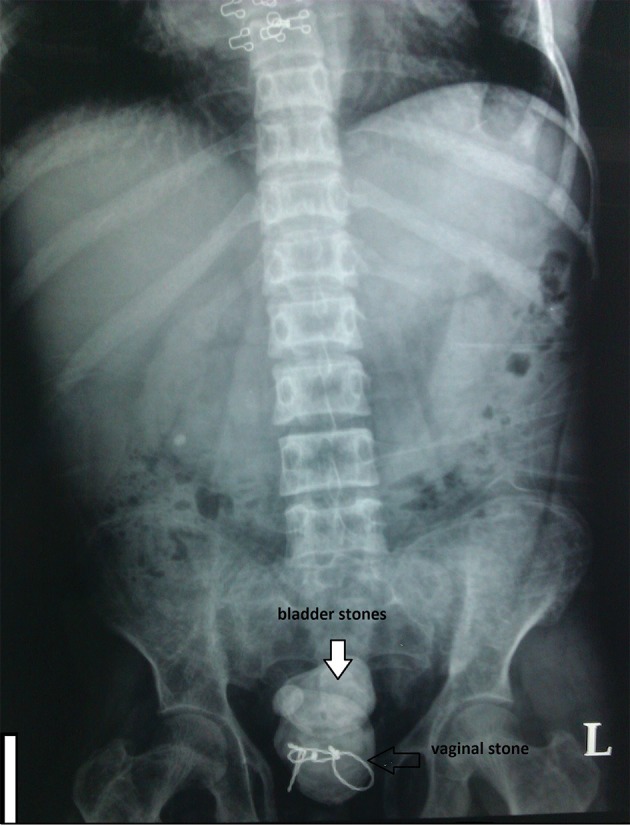
Plain Radiography of the Pati which Shows Bladder Stones and a Huge Vaginal Stone

A 26-year-old woman with previous operation of bladder exstrophy presented supra pubic pain, bloody vaginal discharge and dysuria since about one year ago was treated only medically. She was a case of bladder exstrophy and underwent bladder reconstruction when she was 5 years old. She had been operated 21 years ago in another country and we couldn’t provide her detailed past surgical history. She was on hemodialysis since 12 years ago because of ESRD (end stage renal disease). Her urine volume had gradually decreased in the past 12 years and she was extremely oliguric since about 2 years ago. She also had urinary incontinency but since she was extremely oliguric her incontinency was not prominent. Her physical examination showed a low midline incision of her previous surgery and an anteriorly displaced vagina with bifid clitoris. The patient was a candidate for renal transplant, and her preoperative evaluation abdominal radiograph had been taken. Her radiography showed large stones in the bladder that one of them was around the metallic wire used for fixation of the symphysis pubis in her previous surgery (Figure 1). Her ultrasonograghy showed multiple bladder stones with no report of vaginal stone. So she was admitted and prepared for surgery. Her pre-operative lab data showed: BUN 35 mg/dL, creatinine 5.2 mg/dL, K 5.3 mEq/L, Na 140 mEq/L, blood WBC 8700 (62%PMN, 21%MN and 15.7% other) and U/C: *E. coli* greater than 105 CFU/mL.

**Figure 2.**
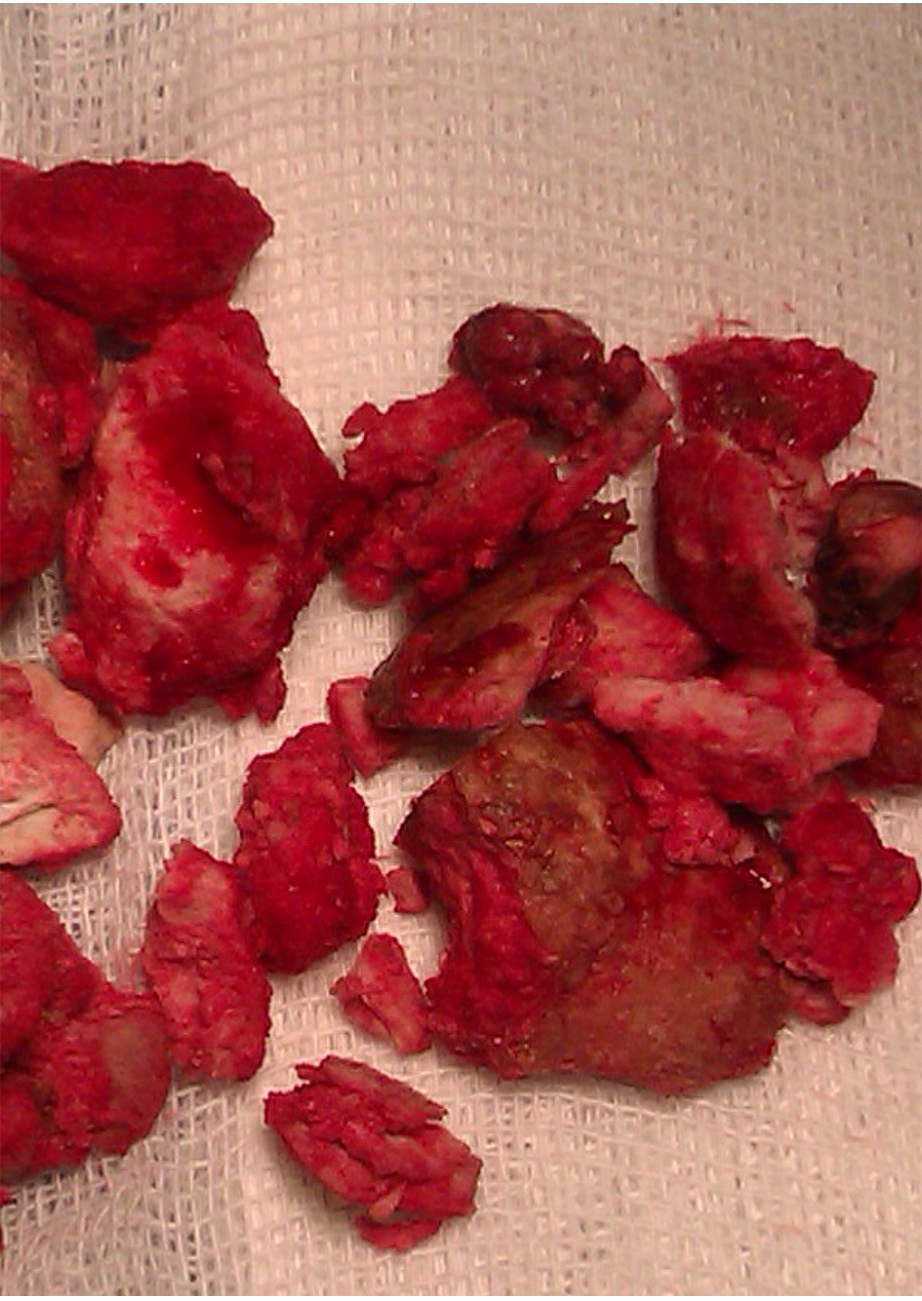
The Stones That Removed From the Bladder

**Figure 3.**
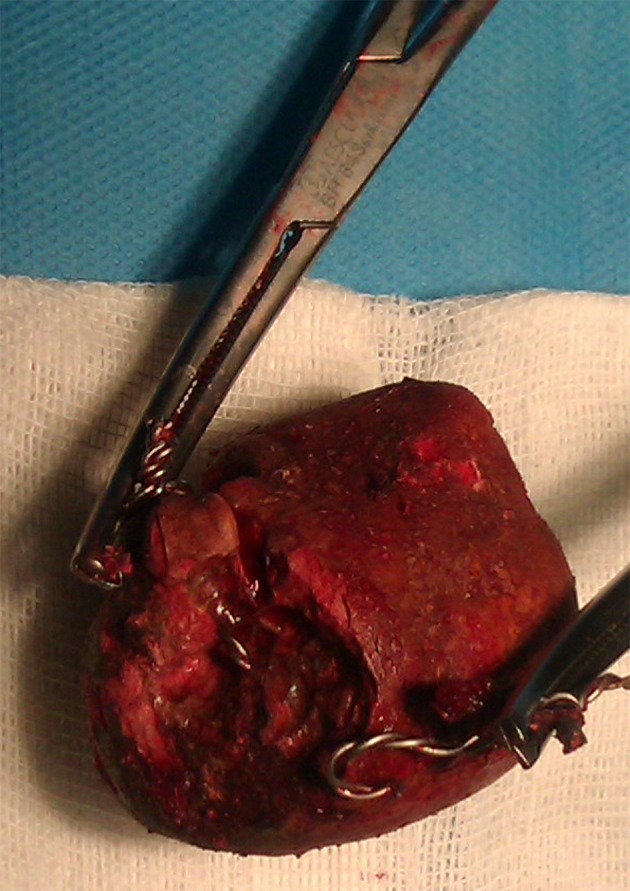
The Huge Vaginal Stone Removed From the Vaginal Cavity

**Figure 4.**
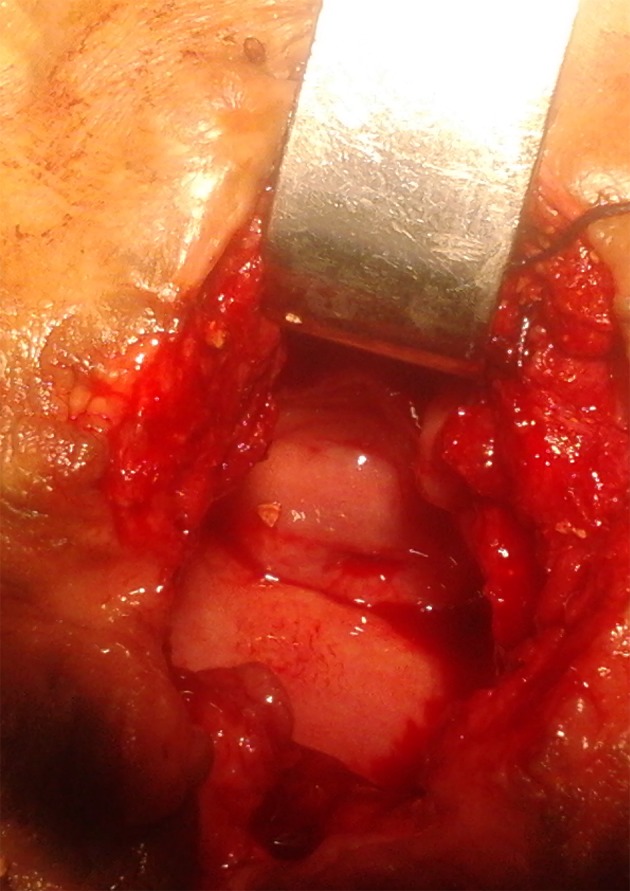
Vaginal Cavity After Removing the Stone and Irrigation. The Cervix of Uterus Is Evident

In preoperative cystoscopy the bladder was full of multiple stones and not suitable for cystolitholapaxy. So we opened the abdominal wall with a short low midline incision and after opening the bladder removed the bladder stones (Figure 2). After that we also palpated another stone which was located behind the bladder in her vaginal cavity. The stone was around the metallic wire and because of its connections appeared unremovable. We cut the wire and then removed the large stone located there (Figure 3). A great amount of pus exited from the vaginal cavity after removing the stone, so we irrigated there with normal saline until no evidence of pus was observed (Figure 4). She didn’t have vaginal fistula. After stone removal and irrigation, the vaginal cavity collapsed and we simply repaired the introitus with simple absorbable sutures. Then we put a Foley catheter in the bladder and reconstruct the bladder and abdominal layers anatomically. She was treated with intravenous antibiotics after surgery. After 20 days her urine culture was sterile. The patient was good in her post-operative period and then was referred to kidney transplant team.

## 3. Discussion

Calculi composed of urinary salts rarely occur outside the urinary tract (6). In females such calculi occurring in the vagina are uncommon and the diagnosis is difficult, so they are often mistaken for bladder calculi (7). Urethrovaginal fistula is the main etiology of most vaginal calculi and the other etiologies are extremely rare, so primary, struvite calculi are the most reported cases in the literature and are often associated with vesicovaginal fistulas (2, 6).

Vaginal stones are classified as primary or secondary. Primary vaginal calculi result from the stasis of urine in the vagina, but secondary vaginal calculi originate from crystallization of urinary components around a foreign body in the vagina (3). Primary vaginal stone is often associated with urinary vaginal fistula, ectopic vaginal ureter, neurogenic bladder, or partial vaginal outlet obstruction. Also infection with bacteria that produce urease can alkaline the PH of the vagina which can predispose the precipitation of struvite calculi (3).

Secondary calculi are less common and often form around a foreign body retained in the vagina such as non-absorbable sutures used in the repair of vesicovaginal fistula (3). Also secondary vaginal calculi can be rarely observed from migration of vesical calculi because of an ulceration of the vesicovaginal septum (6).

The diagnosis of vaginal stones may be difficult because the formation of calculi is slow and usually does not cause any symptoms in the patient. Therefore, when the diagnosis is in doubt, plain radiography of the pelvis should be always taken (3). On the other hand bladder exstrophy is a rare condition occurring one in 25,000-50,000 live births with male to female ratio ranging from 1.5-5 to 1 (4). It is a congenital anomaly characterized by absence of the anterior wall of the bladder, and wide separation of the pubic symphysis (5). Genital defects in females are similar to those of the males, so the mons pubis, both hemiclitori and labia are separated and the vaginal orifice is displaced anteriorly. The reconstruction of genital defects in females is done more easily than in males (8). Surgical correction most commonly consists of primary closure of the bladder followed by bladder neck reconstruction and bilateral ureteroneocystotomy. Closure of the bladder is often associated with pubic osteotomy. Also, it may be necessary to exteriorize the vagina and reconstruct the external genitalia. Long term complications may include urinary incontinence, recurrent urinary tract infections and urolithiasis (5). Urolithiasis is common in patients with bladder exstrophy, occurring in 16% of those with classic exstrophy. It may be related to risk factors associated with surgical reconstruction but the role of metabolic abnormalities is unknown. Although standard treatment of urolithiasis in these patients is effective stone recurrence remains a significant problem. To minimize the stone recurrence, urine chemistry data may provide useful information (9).

Vaginal calculi in these patients are extremely rare and in our Medline search we found only one case report in this context. Primary vaginal calculi are more common than secondary calculi in these patients and may be the result of pooling of urine in a narrow anterior vagina (5).

In our case the remaining metallic wire used in previous reconstruction of bladder exstrophy predisposed the patient to make a huge vaginal stone. It appears that chronic pooling of urine in the vagina gradually caused secondary vaginal stone.
